# Detecting iodine deficiency risks from dietary transitions using shopping data

**DOI:** 10.1038/s41598-023-50180-7

**Published:** 2024-01-10

**Authors:** Roberto Mansilla, Gavin Long, Simon Welham, John Harvey, Evgeniya Lukinova, Georgiana Nica-Avram, Gavin Smith, David Salt, Andrew Smith, James Goulding

**Affiliations:** 1 N/LAB, Nottingham University Business School, Jubilee Campus, University of Nottingham, Nottingham, UK; 2 Division of Food, Nutrition and Dietetics, School of Biosciences, Sutton Bonington, University of Nottingham, Nottingham, UK; 3 Future Food Beacon of Excellence and School of Biosciences, Sutton Bonnington, University of Nottingham, Nottingham, UK

**Keywords:** Risk factors, Public health, Nutrition disorders

## Abstract

Plant-based product replacements are gaining popularity. However, the long-term health implications remain poorly understood, and available methods, though accurate, are expensive and burdensome, impeding the study of sufficiently large cohorts. To identify dietary transitions over time, we examine anonymised loyalty-card shopping records from Co-op Food, UK. We focus on 10,626 frequent customers who directly replaced milk with alternative milk. We then use product nutritional information to estimate weekly nutrient intake before and after the transition. 83% who converted to alternative milk saw a fall in iodine (44%), calcium (30%) and vitamin B12 (39%) consumption, with 57% reducing iodine purchase by more than 50%. The decline is even higher for those switching dairy and meat products. Our findings suggest that dietary transitions - such as replacing milk with alternative milk - could lead to nutritional deficiencies, notably iodine, which, if not addressed, may represent a significant public health concern, particularly in countries which do not mandate salt iodisation.

## Introduction

The rise in popularity of plant-based diets across Europe has been a notable trend in recent years, driven by concerns over environmental sustainability, animal welfare, and personal health^1,2^. As an increasing number of individuals make the transition to plant-based eating patterns, there is a growing need to understand the potential nutritional changes and health outcomes associated with such dietary shifts, particularly for vulnerable groups, including women of childbearing age, children and the elderly. Notably, the rapid and sustained loss of a significant proportion of dietary iodine can hold grave implications. While the potential benefits of plant-based diets have been widely recognised^3^ there are certain aspects, such as iodine intake and its health implications, that remain less understood.

Iodine is an essential dietary mineral required for the synthesis of thyroid hormones and is consumed in the form of iodised salt by much of the global population due to salt iodisation being mandated by many governments^4^. In the UK, however, this is not the case, and consequently, almost all iodine intake is from animal products, with the principal source being milk^5,6^. For some, milk contributes up to 70% of their daily intake^7^, with additional sources including dairy products and seafood. Iodine is supplied to cows via fortified feed and mineral supplements, as well as indirectly by using iodinated teat disinfectants from which the animal can absorb iodine^7^. Circulating iodine is actively pumped into the milk by the mammary tissue as it is required by the developing calf for its thyroid hormone synthesis.

Iodine deficiency has serious potential consequences, including goitre and hypothyroidism. Whilst the impact of iodine deficiency on thyroid hormone signalling may take time to manifest clinically in adults, for a developing fetus, even relatively mild deficiency can lead to impairment of growth, skin development and sexual maturation, and more severely, cognitive dysfunction or in extreme cases even cretinism, stillbirth and fetal resorption^7^.

The daily requirement for iodine intake in adults is 140 μg (UK) to 150 μg^8^, increasing to 250 μg Day$^{-1}$ during pregnancy. For milk consumers, daily consumption of 200 - 300 ml of milk per day would yield upwards of 50–75 μg, providing up to 50% of the required intake. As salt is not mandatorily iodised in the UK, those transitioning to dairy-free diets may be at significant risk of chronic underconsumption^9,10^. For many, the transition to a plant-based diet involves the direct replacement of milk, dairy and seafood with “equivalent” style products, the majority of which are not currently fortified with iodine^5,11^. Recent indications of iodine status in the UK suggest that levels of intake, whilst adequate at the population level are, however, low for a significant number of people, with around 10% of the total population recorded in the years 2016-2018 of the national diet and nutrition survey (NDNS), consuming below the Lower Reference Nutrient Intake (LRNI; that amount which is sufficient for only  2.5% of the population), with 26% of teenagers falling into this group^12,13^. The proliferation of unfortified milk and dairy alternative products since 2018 may have further impacted intake levels, resulting in a larger proportion of the population consuming below the LRNI.

Thus, there is a significant need to identify groups at particular risk of chronic iodine deficiency. However, this is challenging to assess accurately by traditional methods, as sample sizes available to research are typically small, in the order of tens or very occasionally hundreds of participants. This poses significant problems when attempting to extrapolate outcomes of dietary transition to a wider population. To overcome this, we explore dietary changes via anonymised shopping data records logged by the Co-op Food brand, a major UK food retailer with over 2000 convenience stores and supermarkets and the greatest geographical coverage of any UK food retailer with over 95% of the population living within 5 miles of a store. Specifically, we answer the research question: How do sources and overall shopper dietary intake of iodine vary when transitioning from milk to alternative milk options?^14^

## Methods

Strictly anonymised data covering store transactions over 2.5 years (July 2019–December 2021) for over 4 million co-op members was filtered using predefined exclusion criteria to identify representative frequent customers making significant food purchases (i.e. anonymised accounts whose purchases are likely reflective of their overall consumption patterns). This process identified more than 500,000 frequent shoppers. This sample was then used to establish shopping trajectories where logs exhibited dietary transitions during the study period, specifically regarding milk and dairy exclusion, finding 10,626 applicable accounts. A nutritional mapping exercise was then performed to link nutritional data to the retailer’s grocery product portfolio using a combination of public data sources and the retailer’s in-house nutritional database. This process focused on matching the most popular and iodine-rich food products to ensure that estimated iodine intake levels could be confidently measured. Around 90% of total sales volumes and customer spending were matched to their nutritional content. This represented >99.9% of sales of iodine-rich foods (i.e. seafood, dairy and eggs) for the sample.

The nutritional profile of the sample was then analysed across the pre- and post- dietary transition points - in this instance, when an individual stopped purchasing dairy milk and started purchasing plant-based milk. A minimum threshold of 4 weeks of purchases, both pre- and post- transitions were required to ensure confidence in the customer’s purchasing behaviour. A further two categories of dietary transition were added to separate customers who made additional changes to their diet, i.e. customers who also switched to other plant-based dairy products (e.g. vegan cheese) and customers who replaced meat and poultry products with plant-based versions.

Average weekly iodine levels of grocery purchases were calculated and analysed to examine any reduction that resulted from the transition to plant-based milk. In addition, the effects of the dietary transition on other key nutritional components of dairy milk, including calcium, vitamin B12 and saturated fat, some of which were fortified in plant-based milk, were also analysed to compare the impacts of transition on other nutritional elements. Statistical significance of the differences in nutrient intakes between the mean ones before and after the transition to plant-based products was assessed using the Paired-samples sign test. The results of this analysis are described in detail in the following section.

### Transactional data

The study utilises historical transaction (purchase) data from the member card scheme of Co-op Food, one of the major grocery retailers in the UK. The retailer rigorously anonymised and handled the data following the UK’s strict confidentiality, privacy, and established ethical norms and requirements. With a national scope, the data set contains the grocery purchases of over 4 million consumers between 2019 and 2021. For each purchase, the data details ‘when’, ‘where’, ‘what’, and ‘how much’ each individual bought. Similarly, for each item purchased, it was possible to identify descriptors, such as the ‘quantity’, ‘price’, ‘item description’, ‘size’, and the ‘product category’.

To investigate within-individual diet transition and nutrient intake levels, the study only considers customers with repeat food purchasing behaviour at a level reflective of overall food consumption patterns to ensure minimum basket and spend levels. To achieve this, we establish strict inclusion criteria to extract *frequent customers* from the initial data set. These inclusion criteria are more stringent than those outlined in comparable studies utilising large transactional data sets to ensure representativeness (e.g.^15^,). The inclusion criteria applied to identify *frequent customers* are as follows:

- At least one weekly shopping visit for at least six consecutive months.
- Average spend per basket greater or equal to $\pounds$5.
- Average spend per week between $\pounds$5 and $\pounds$400 (inclusive).
- An average of less than 14 baskets a week to eliminate outlying behaviour.

A total of 512,397 *frequent customers* meet the inclusion criteria, with more than 992 million items sold and 200 million transactions (number of purchases/baskets). This sample is further filtered below.

### Products’ nutrition information

Linking food product data to their nutritional content is a non-trivial task. There are over 31,000 food products contained in the retail data set used in this study. Each product has a text string called *item description*, which typically includes the brand name, product name, and product size (in that order). However, not all item descriptions exactly follow this pattern. Products are also categorised into a three-level hierarchy based on their *department*, *section*, and *subsection*. This is the only information available to match the products to their nutritional content.

Three data sources were used to map grocery products to their nutritional content. The primary data source used in this study was the UK Composition of Foods Integrated Data set (CoFID), also often referred to as McCance and Widdowson (the authors of the data set). The secondary data source was the retailer’s brand nutritional data set provided with the data. The third data source was the online nutritics.com data set. Where these data sources were incapable of providing nutritional data for a product that was identified as a significant source of Iodine and/or was sold in significant volumes to our customer sample, then the product’s nutritional content was manually calculated based on the product ingredient list (identified from a web search).

CoFID was this study’s primary nutritional data source as it includes detailed data on micronutrients, including iodine. The retailer’s nutritional data only included macronutrients and selected micronutrients, which did not include iodine. For this reason, products were only matched to the retailer nutrition data where a CoFID nutritional match could not be established.

A three-stage method for matching nutritional data to product data was employed in this study. This process was performed for each of the three nutritional data sources in order of their primacy (i.e. CoFID, retailer nutrition, Nutritics). The three stages of nutritional mapping were:

1. Product and nutritional data sets were imported to a PostgreSQL database. The PostgreSQL *similarity* function, which performs fuzzy text matching using trigrams, was used to compare the item descriptions in both data sets and return those with a similarity above a specified confidence level. This level was initially set to 0.9 and then iteratively reduced to 0.5 to maximise the number of matches. Matches from each iteration of the confidence level were manually verified. Verified matches were added to a nutritional look-up table.
2. Unmatched products after step (1) were then matched using the *department*, *section*, and *subsection* fields in the product table. These fields were compared to the food description in the nutritional data source(s) using the PostGresSQL *similarity* function. Where verified matches were identified by this method, all products in the matched *department*, *section*, and *subsection* were added to the nutritional look-up table.
3. Food products still unmatched after steps (1) and (2) were then manually matched using their sales volume to prioritise matching, i.e., highest-selling products were matched first. This process was repeated until a specified sales volume threshold was reached.

Where the last step of the matching process cannot find a matching food in all three nutritional data sources, then the unmatched products were flagged for manual calculation of their nutritional content based on their ingredients, where such data was available via a web search.

Just over a third of the total food products in the retailer’s data set were matched to the nutritional data sources based on their sales volumes. Overall, this represented around 90% of total sales quantity and spending on all food items in the retail data set.

For this study, nutrition matching prioritised food products likely to contain significant levels of iodine (e.g. food items containing seafood, milk, dairy and eggs). A threshold of 100 sales was set over the 30 months the data covered. All iodine-rich products with sales above the threshold to our frequent customers that remained unmatched after the initial mapping exercise were reevaluated and matched manually. For these foods, 90% of products, representing 99.9% of sales, were matched to their nutritional content. This ensures high confidence in our assessment of dietary iodine intake levels from the retail data.

### Experimental design

We establish a ‘transition point’ approach to identify nutrient changes occurring across plant-based dietary transitions. For our analysis, we define the transition point as the date a consumer ceases purchasing a particular product category, such as milk and dairy items and begins or continues purchasing plant-based equivalents. Given that the primary focus of this study is to analyse iodine ingestion levels, we propose using milk as the product category that determines the transition point for each customer. This is because milk is the primary source of iodine, is widely consumed, and is affordable. Thus, the transition point for each consumer will be the date they cease purchasing milk and begin or continue purchasing plant-based milk (e.g., almond, soy, oat, and coconut). Therefore, to establish this transition point, we extracted 168,123 consumers who purchased both milk and plant-based milk during the purchasing period from the 512,397 *frequent customers*.

To assess the impact of shifting to plant-based milk on the consumers’ nutrient consumption levels, we examined the minimum number of weeks for which milk purchases were consistently made before the transition point and matched that with the same number of weeks that plant-based milk purchases were made after the transition point.

We determined the minimum number of weeks by analysing the decrease in the sample size, in this case, the number of *frequent customers*. Overall, increasing the number of comparable weeks before and after the transition significantly reduces the sample size. There is no evidence in the literature to establish an optimal number of minimum weeks to evaluate nutrient intake levels. However, based on our analysis, a minimum of four weeks is a judicious period to examine such variations. With this inclusion criteria, we obtained a sample of customers replacing milk of 10,626 *frequent customers*. A previous systematic review suggested that among loyal customers, supermarkets may account for between 63% and 67% of total household food expenditure^16^). According to the UK’s ’Living Costs and Food Survey’, the average UK household food expenditure per week was around $\pounds$62–63^17^. Using these estimates, we therefore suggest that, on average, UK households likely spent $\pounds$39.06 per week (63% of $\pounds$62) at supermarkets during the study period. For the Co-op Food cohort reported within this study, the average weekly spend was $\pounds$33.97, likely representing around 55% of their total household food expenditure.

Within the 10,626 customers, we identified two other groups to analyse. First, a sub-sample of consumers who, besides replacing milk at the transition point, also substitute dairy products with plant-based alternatives. Similarly, a second sub-sample of consumers who, in addition to milk, replaced meat and poultry products with plant-based options at the transition point. The nutrient variances of these groups are compared to the recommended intake levels and the nutrients from customers with a complete omnivore diet throughout the whole data set period sampled from the 512,397 *frequent customers*. Though we note that dietary reference values are risk assessment tools, not diagnostic reference points. The sample size (N) for each group is shown in Table 1.

### Assessing iodine intake from shopping data

To evaluate weekly iodine intake before and after the transition point, we aggregated each customer’s transaction record by week. This enables us to calculate the total quantity of iodine associated with weekly purchases before and after the transition point. To achieve this, we utilised the iodine μg per 100ml from each product’s nutrition information (collected and linked to the transaction data as described in section "Products’ nutrition information") to compute the total μg of iodine per product format. We calculated the average weekly iodine intake at three levels:

1. *Customer-level*: We compute each customer’s average weekly iodine intake by adding up the total amount of iodine consumed weekly before and after the transition point. Subsequently, we determined the mean iodine intake for each period per customer.
2. *Population-level*: We calculate the mean of all customers’ average weekly iodine before and after the transition point.
3. *Population-level per week*: We suggest setting the week of each customer’s last milk purchase (referred to as the transition point) as week zero. By considering purchases made exclusively during the weeks immediately preceding and following this point, we calculated the average iodine intake for each of those weeks.

As per the paper’s title, this study focuses on identifying risks of iodine deficiencies caused by dietary transitions to plant-based milk. To contextualise the impact of this dietary transition, we also calculated the average weekly intake of other nutrients significantly present in dairy milk products: calcium, B12, and saturated fats, following the same methods described above. By calculating the changes in nutrient consumption at different levels, we aim to estimate changes in these nutrient intake patterns before and after the transition point to highlight the level of iodine deficiency caused by the transition.

The values calculated for these nutrients are subject to less certainty than iodine levels since the nutritional mapping exercise was primarily tailored to iodine-rich products. However, given the high threshold of nutritional mapping achieved, we still have high confidence in their estimated intake levels.

### Statistical analysis

We used Python 3.9.7 to conduct exploratory analyses of the variation in iodine, calcium, B12, and saturated fats before and after the transition to plant-based alternatives at various levels. The significance level for all statistical analyses was set at $P \le .001$ (two-tailed). We examined the normality of the average weekly nutrient intakes using the Shapiro-Wilk test for normality. Iodine, calcium, B12, and saturated fats intake values were not normally distributed. Therefore, we checked if the distributions of differences between the consumers’ average weekly nutrient intakes (paired observations) were symmetrical in shape using the skew test and density plots for visual examination. The statistical significance of the differences in nutrient intakes between the mean nutrient intakes before and after the transition to plant-based products was evaluated using the Paired-sample sign test, with all necessary assumptions being met. All tests were done via the Python statistical library Scipy.

## Results

### Iodine deficiency risks from dietary transitions

The primary sample to be analysed consists of 10,626 frequent customers who met the inclusion criteria (outlined in section "Transactional data") and who purchased milk for at least four weeks before the transition point and plant-based milk for at least four weeks after the transition. This sample contains over 19 million items and 3,168,283 million baskets (transactions) sold.

General statistics of the consumers’ average weekly iodine intake before and after switching to plant-based products for all dietary groups are shown in Table 1. The dietary group that just replaced milk had a mean iodine level of $345\pm 229$ $\mu g/week$ before the transition, which decreased to $194\pm 166$ $\mu g/week$ after the shift, indicating a 44% percentage change ($p <.001$). The dietary group replacing milk and dairy with their plant-based equivalents also saw a percentage change of 63%. Before the transition, the mean iodine level of this dietary group was $312\pm 226$ $\mu g/week$, while after the transition, it was $116\pm 148$ $\mu g/week$. The dietary group that switched from milk and meat & poultry to plant-based alternatives (but continued to eat other dairy products such as cheese and yoghurt) shows the lowest percentage change of 40% ($p <.001$), with a mean iodine level of $229\pm 194$ $\mu g/week$ before the transition and a mean of $176\pm 155$ $\mu g/week$ after the shift.

Customers bought an average of 4 to 5 items per transaction throughout the analysed period. Thus, the decrease in iodine levels is not due to a drop in the number of items purchased but rather a lack or insufficient amount of iodine in plant-based products. The baseline dietary group that followed an omnivore diet and did not experience any major dietary change during the study period showed a mean iodine level of $680\pm 445$ $\mu g/week$. Although this mean iodine level is still below the Reference Nutrient Intake (RNI) of between $980 - 1050$ $\mu g/week$, it is around 50% higher than the three dietary groups mentioned previously. Calcium, vitamin B12 and saturated fat results are also detailed in Table 1 following similar patterns.

**Table 1:** Average weekly nutrient statistics before and after transitioning to plant-based products.

N	Food group(s) excluded	Measure	Iodine (μg)			Calcium (mg)			B12 (μg)			Saturated fats (g)		
			Before weekly Avg.	After weekly Avg.	Percentage change	Before weekly Avg.	After weekly Avg.	Percentage change	Before weekly Avg.	After weekly Avg.	Percentage change	Before weekly Avg.	After weekly Avg.	Percentage change
10,626	Milk^a^	Mean	345	194	44%$$^{***}$$	2082	1449	30%$$^{***}$$	12.8	7.8	39%$$^{***}$$	93.0	72.6	22%$$^{***}$$
		SD	229	166		1168	994		7.9	6.1		61.1	57.5	
		Median	294	152		1852	1,221		11.1	6.2		78.2	57.6	
		Q1	178	79		1230	737		7.0	3.7		51.1	33.3	
		Q3	456	263		2691	1914		16.8	10.3		118.9	95.1	
106	Milk^a^ and Dairy^b^	Mean	312	116	63%***	1946	1108	43%***	11.8	5.1	57%***	63.7	25.1	61%***
		SD	226	148		1026	647		7.8	3.6		49.4	32.7	
		Median	254	71		1765	1023		9.9	4.1		51.2	17.3	
		Q1	155	10		1208	607		6.1	2.7		32.3	9.1	
		Q3	430	166		2505	1496		16.6	6.9		79.1	28.1	
367	Milk^a^ and Meat^c^	Mean	292	176	40%***	2032	1553	24%***	10.6	6.6	38%***	71.3	52.5	26%***
		SD	194	155		1055	1005		6.3	4.9		48.5	45.9	
		Median	252	135		1909	1364		9.4	5.3		60.1	39.4	
		Q1	143	60		1213	828		6.1	3.3		39.6	21.2	
		Q3	408	240		2676	2010		13.9	8.3		91.0	70.3	
500,256	Omnivore	Mean	680			3795			22.6			175.4		
		SD	445			2277			14.6			108.1		
		Median	586			3321			19.6			150.1		
		Q1	354			2172			12.1			101.1		
		Q3	898			4875			29.7			221.1		
Recommended weekly intake			980−1050			4900–5000			10.5–11.5			140–210		

***p$<.001$, paired-samples sign test.

^a^Customers have replaced milk with plant-based milk.

^b^Customers have replaced dairy with plant-based dairy items.

^c^Customers have replaced meat with plant-based meat & poultry items.

**Figure 1:**
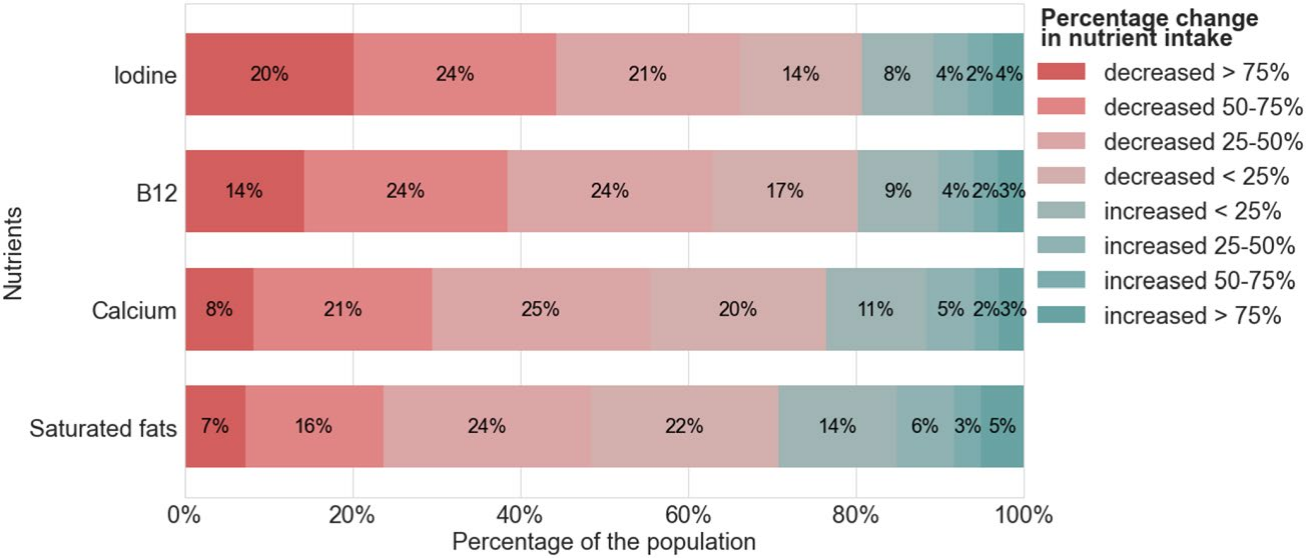
The proportion of the total customers grouped by the percentage of change in nutrient intake.

The average weekly intake of iodine at the population level grouped by the percentage of change is presented in Figure 1, which also displays the results for calcium, vitamin B12 and saturated fat. More than 81% of the customers decreased their average weekly intake of iodine once they switched to plant-based milk. Of those individuals, approximately 14% experienced a decrease of less than 25%, but around 20% experienced a severe iodine reduction of more than 75%. The remaining 45% of the individuals experienced a drop between 25% and 75%. Similar results were observed for calcium and vitamin B12. In contrast, more than 71% of consumers showed a positive reduction in their weekly intake of saturated fat, with about 23% reducing over 50% of their weekly intake.

**Figure 2:**
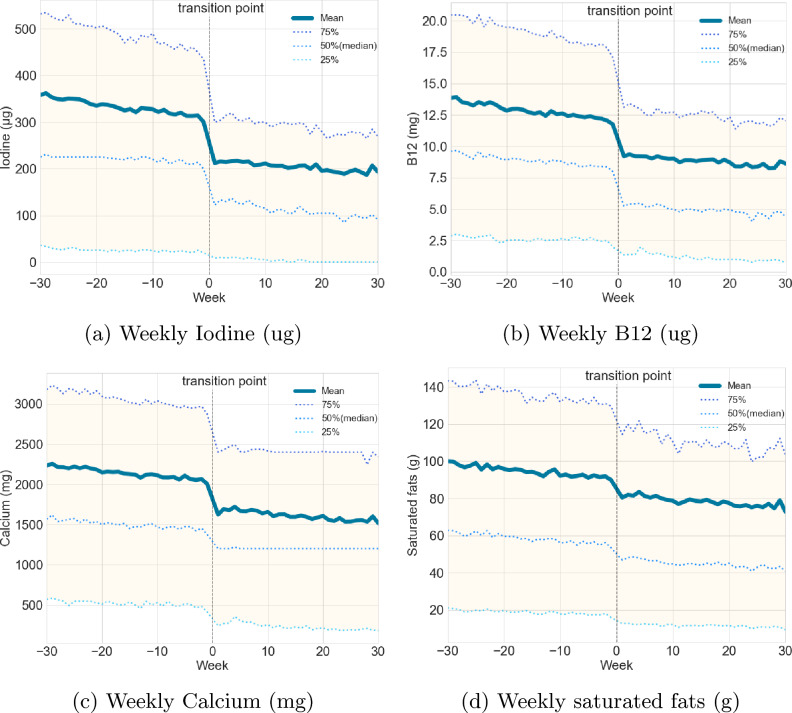
Comparison of the average nutrient intake 30 weeks before and after replacing milk with plant-based versions.

The average iodine intake aggregated per week at the population level is illustrated in Figure 2a. After harmonising all individuals’ transition points (see section "Assessing iodine intake from shopping data" for explanation), a total of 30 weeks before and after the shift were aggregated to evaluate the temporal variation of the average iodine intake levels. For the weeks before the transition point, the mean iodine intake fluctuates between 301 $\mu g/week$ and 362 $\mu g/week$, while for the weeks following the transition, it drops drastically to a range between 187 $\mu g/week$ and 217 $\mu g/week$. This reflects a general decrease of approximately between 38% to 40%. A similar decrease was seen for vitamin B12 of between 29% to 33%, and for calcium between 10% to 25%, while for saturated fats, a positive decrease of around 16% overall (see Fig. 2b,c,d).

**Figure 3:**
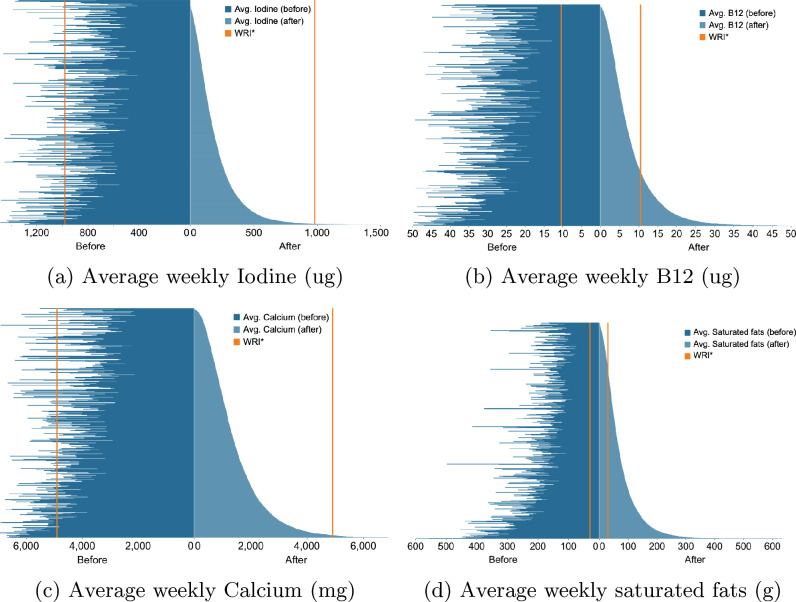
Comparison per customer of the average weekly nutrients before and after replacing milk with plant-based milk.

Figure 3 illustrates the variation in the average weekly nutrient intake per customer before and after the transition for the entire sample of 10,626 customers who switched from milk to plant-based alternatives. Figure 3a illustrates a descending order of iodine variation experienced by consumers following the transition, with those having the most significant nutrient decrease at the top and the others with lesser decreases below. Additionally, it displays the changes in vitamin B12, calcium, and saturated fats. After switching to plant-based milk, most consumers (around 80%) reduced their iodine intake, bringing them further away from the RNI. Consequently, more than 90% of the customers were below the RNI after the change.

A similar result can be seen for calcium and vitamin B12, with most customers experiencing a significant drop after the switch. A majority of them, over 90%, do not meet the recommended RNI value for calcium post-transition. As for vitamin B12, most customers were meeting the RNI before the switch, but around 60% of them now consume less than the recommended amount.

Finally, prior to transition, people purchased an average of $1597\pm 640$ *ml*/*week* of milk, with a median of 1458 *ml*/*week*. This declined after the transition, with the average amount of plant-based milk purchased was $1226\pm 394$ *ml*/*week*, with a median of 1000 *ml*/*week*.

## Discussion

The data highlight two notable findings. First, as expected, the cessation of milk purchasing led to a considerable reduction in the quantity of iodine purchased. At present, many manufacturers of plant-based milk have not fortified their products with iodine. Eight of the top ten highest-selling plant-based milks in this study were unfortified. Second, the volume of milk removed during the transition was not replaced by equivalent volumes of plant-based milk. In part, this is likely because of the difference in the average price per litre of milk ($\pounds$1.01) and plant-based milks ($\pounds$1.70). This reveals that people who choose to exclude cow’s milk, almost universally, will significantly reduce their iodine intake even when choosing fortified products, as the volumes consumed do not reach those of their prior milk consumption. Indeed, those people who do switch between milk and plant-based milks are already likely tapering their intake of milk in advance of the switch (as is illustrated by the ’before weekly average’ difference between the milk switching and omnivore group), thus indicating the long term decrease is likely even more pronounced. This finding is further supported by observing a concomitant drop in vitamin B12 and calcium, even though these are typically fortified in milk replacements. This poses a public health challenge which may not be overcome by fortification of milk replacements alone, necessitating alternative routes of provision for people excluding milk.

As plant-based milk is drank in smaller volumes than milk on average^18^, fortifying additional plant-based dairy replacements may be a sensible public health strategy. The results show that those who excluded dairy in addition to milk had the lowest overall intake of any group in the cohort. This presents an opportunity for manufacturers of plant-based dairy replacements (e.g. vegan cheeses, creams, butter, and spreads) to fortify their products with equivalent amounts of iodine present in animal-derived forms.

The methodology proposed in this study has the potential to identify broader impacts of dietary transitions at a national scale, such as the risk of mineral deficiencies, as highlighted here, particularly where shoppers choose plant-based substitutions. We note that the approach has a number of key strengths, including: the scope for large sample sizes; consistency of data collection for all participants; no bias due to under- or over-reporting; and detailed product descriptions unlike those typically present in diet diaries. The approach is transparent, allowing the retail sector to analyse temporal changes to micronutrient purchasing as people adopt and abandon specific diets over time. Historically, tracing dietary restrictions and investigating their effect on health and well-being across a large population has been challenging. Previous findings in this area have been achieved using self-reported surveys and small-scale studies, which can suffer from low accuracy or bias. However, it is important to note that large-scale dietary surveillance achieved through shopping data is not a panacea and works best when designed to corroborate the findings of traditional methods.

Despite providing unprecedented insight into the comparative acquisition of iodine-rich products, this anonymised study makes no claims about the demographic makeup of the cohort. While the loyalty card data from Co-op Food represents a reliable data source, it is important to acknowledge that not all individuals use loyalty cards, and some may use multiple cards. This may affect the generalisability of the findings. Retailers are in a position to know the respective demographic segments that constitute those making dietary shifts, and future research should aim to make this data public in a way that preserves individual shopper privacy. For example, pregnancy often causes changes in food consumption behaviour, particularly due to reductions in fish and/or dairy products for perceived health reasons, while at the same time, the recommended daily iodine intake actually increases for this group. Knowing the demographics of those exhibiting transitions in greater detail would enable targeted interventions to ensure more focused means of fortification, particularly where the risk of iodine deficiency may be especially pronounced.

Additionally, we note that purchasing food will not necessarily result in the food being consumed by the same person. The inclusion criteria for the study strictly ensure only regular customers who frequently purchase a high portfolio of food from Co-op Food are selected for the cohort, but information regarding who is consuming the products is impossible to ascertain (particularly where households might have mixed composition). In this light, the post-transition iodine intake statistics emerging from our analysis appear even starker - results already compare unfavourably to the recommended daily intake for a single person; if, in some cases, accounts are in actuality reflecting consumption across multiple individuals, then the situation worsens further. Further analysis is required here, and an increased understanding of household composition associated with loyalty card usage would provide deeper insights into the impacts of dietary transitions and the role of social influence in switching behaviour^19^. Additionally, future work may benefit from the adoption of iodine data from multiple product lookup databases, and by paying further attention to the provenance of different products.

Existing research on micronutrient security often focuses on the role of affluence and deprivation as a cause of deficiency. While assessment of the impact of deprivation on iodine intake is beyond the scope of this work, it would be extremely useful for policy to identify whether micronutrient deficiency risks correlate more with cultural shifts around the ethics of eating than they do with the lack of affordable options. Linking indices of deprivation produced by the national government, for example, would likely provide valuable understandings of whether dietary transitions have similar effects across households of different socioeconomic statuses. It may be possible, therefore, to compare whether wealthy households, who may shop at different places, experience similar nutrient deficiency risks after a dietary transition compared to less affluent and less mobile households.

Finally, the observational methodology introduced in this paper offers several advantages and further applications beyond dietary transitions. The method’s ability to simulate ‘what-if’ scenarios provides a potentially valuable tool for a range of situational diagnostics, particularly in resource-constrained contexts like pandemics, by identifying the optimal timing for interventions or revealing when temporal changes at scale are going to cause crises related to the cost of living. As more people change their diets in response to environmental sustainability, animal welfare, and personal health, there must be a commensurate increase in the observation of unintended potential micronutrient deficiency risks.

## Data Availability

The data that support the findings of this study are available from Dr John Harvey (john.harvey@nottingham.ac.uk), but restrictions apply to the availability of these data, which were used under license for the current study, and so are not publicly available. Data are, however, available from the authors upon reasonable request and with permission of Dr John Harvey (john.harvey@nottingham.ac.uk).
